# Tumor cells derived-exosomes as angiogenenic agents: possible therapeutic implications

**DOI:** 10.1186/s12967-020-02426-5

**Published:** 2020-06-22

**Authors:** Mahdi Ahmadi, Jafar Rezaie

**Affiliations:** 1 Tuberculosis and Lung Diseases Research Center, Tabriz University of Medical Sciences, Tabriz, Iran; 2 Solid Tumor Research Center, Cellular and Molecular Medicine Research Institute, Urmia University of Medical Sciences, Shafa St, Ershad Blvd, 1138, Urmia, 57147 Iran

**Keywords:** Extracellular vesicles, Exosomes, Tumor angiogenesis, Mesenchymal stem cells

## Abstract

Angiogenesis is a multistep process and various molecules are involved in regulating it. Extracellular vesicles are cell-derived particles, secreted from several types of cells and are known to mediate cell-to-cell communication. These vesicles contain different bio-molecules including nucleic acids, proteins, and lipids, which are transported between cells and regulate physiological and pathological conditions in the recipient cell. Exosomes, 30–150 nm extracellular vesicles, and their key roles in tumorigenesis via promoting angiogenesis are of great recent interest. In solid tumors, the suitable blood supply is the hallmark of their progression, growth, and metastasis, so it can be supported by angiogenesis. Tumor cells abundantly release exosomes containing different kinds of biomolecules such as angiogenic molecules that contribute to inducing angiogenesis. These exosomes can be trafficked between tumor cells or between tumor cells and endothelial cells. The protein and nucleic acid cargo of tumor derived-exosomes can deliver to endothelial cells mostly by endocytosis, and then induce angiogenesis. Tumor derived-exosomes can be used as biomarker for cancer diagnosis. Targeting exosome-induced angiogenesis may serve as a promising tool for cancer therapy. Taken together, tumor derived-exosomes are the major contributors in tumor angiogenesis and a supposed target for antiangiogenic therapies. However, further scrutiny is essential to investigate the function of exosomes in tumor angiogenesis and clinical relevance of targeting exosomes for suppressing angiogenesis.

## Background

Angiogenesis, rising new capillaries and blood vessels from pre-existing vasculature bed, occurs in physiological status during embryonic development and wound healing, is essential for tumor growth and metastasis [1]. Tumor growth needs blood supply to provide oxygen and nutrients for metabolic functions. This need to be accomplished typically through angiogenesis [2]. Tumor-associated blood vessels are abnormal compared to other organs, they represent abnormal morphology, excessive branch, abundant and bulges and blind ends, irregular endothelial cells (ECs) lining, and defective pericyte coverage and basement membrane [3]. These features arise because of excessive and sustained pro-angiogenic signaling. The ECs of the tumor environment show structural and molecular character that discern them from those of in normal organs [3]. In malignant tumors, tumor cells get invasive activities and make a stromal response including robust angiogenesis [3]. Therefore, tumor development from a benign to a malignant step is classically related with an angiogenic switch, initiating the growth of a vascular bed that is aggressively rising and infiltrative [3, 4]. Angiogenic programming of solid tumors is a multidimensional process planned by tumor cells in concert with different stromal cells and their active products, which comprise growth factors and cytokines, the extracellular matrix and secreted extracellular vesicles (EVs) [5]. EVs are cell-derived vesicles that play the pivotal roles in intracellular communication and also in biological events such as angiogenesis [6]. These vesicles contain different kinds of active biomolecules from donor cells, which affect function, fate, and morphology of target cells [7]. Exosomes released by different cells in the tumor microenvironment have emerged to be a key modulator of cell communication [8]. Most cells produce exosomes, however, tumor cells actively produce exosomes due to presence of stress condition such as hypoxia, which induces exosome biogenesis and secretion [9]. In cancer patients, particularly individuals with progressive or metastatic tumors, have significantly amplified numbers of exosomes in the plasma rather than healthy donors [10]. Tumor cell derived-exosomes can reach to neighboring tumor cells and surrounding cells such as ECs and promote tumorigenesis [6]. The modulation of the tumor microenvironment and the construction of a pre-metastatic niche are the key reprogramming dealings that are mediated by exosomes [11]. Exosomes facilitate tumor angiogenesis and seem to regulate mechanisms involved in vessel growth. In this review, we discuss kinetics of exosomes and also describe recent knowledge available for the roles tumor cell derived-exosomes play in the process of angiogenesis.

### Angiogenesis

**Fig. 1:**
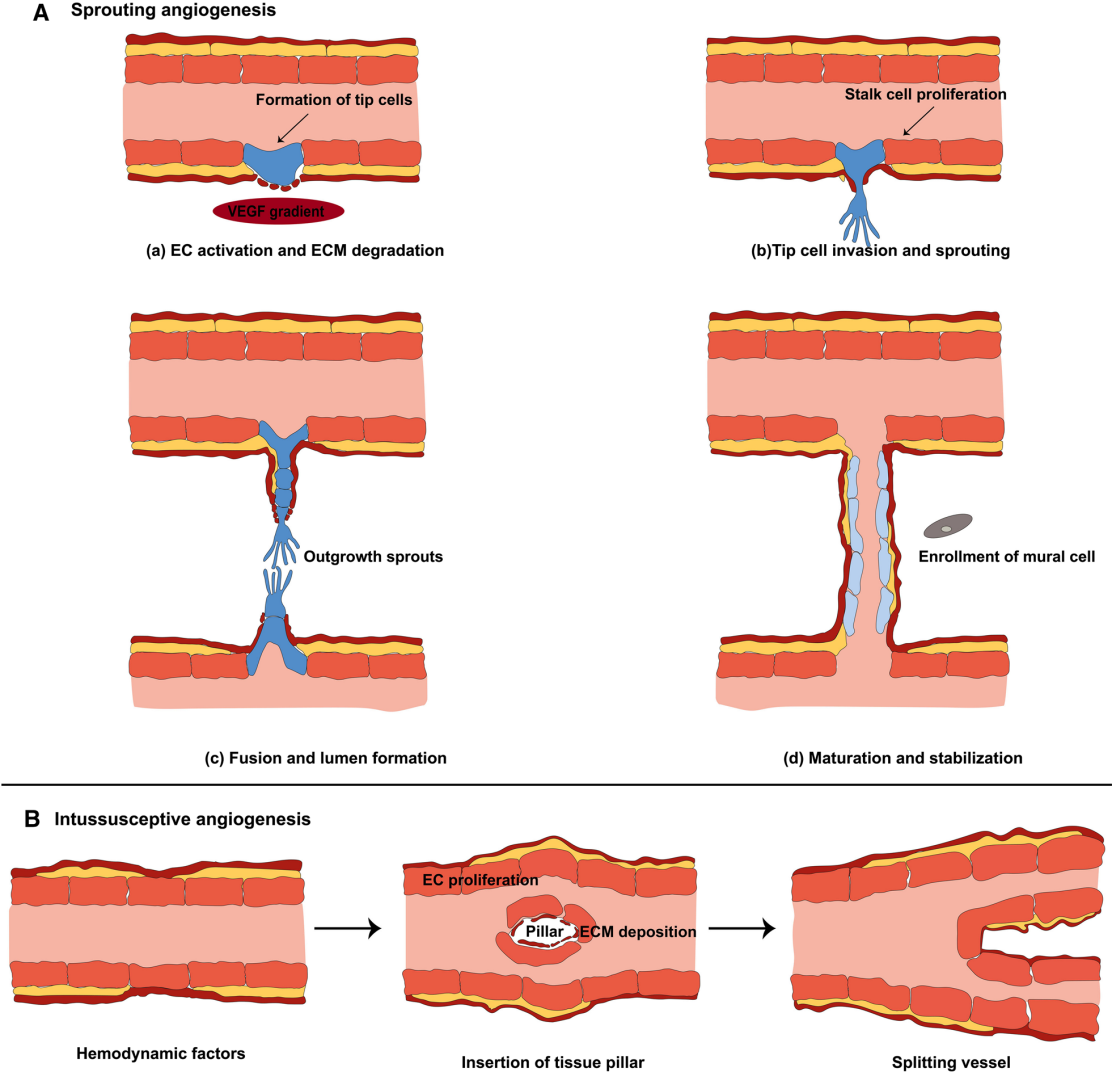
Schematic model of sprouting angiogenesis (**A**). Angiogenesis is regulated by the balance between pro-angiogenic and anti-angiogenic factors. Initiation of angiogenesis requires the degradation of the extracellular matrix and activation of some endothelial cells as tip cells (**a**). As shown, tip cells are characterized by filopodia and migratory behavior toward angiogenic factors such as VEGF, while stalk cells which support formation of new network, are characterized by their high proliferative capacity (**b**). Different sprouts connect with each other by tip cells to form lumen (**c**), and after formation of new vessel, endothelial cells undergo quiescence followed stabilization and maturation by mural cells and the extracellular matrix deposition (**d**). Intussusceptive angiogenesis (**B**). The splitting of vessels through the inclusion of tissue pillars is a divide mechanism that results to the development of blood vessels. The detailed mechnismds involve in this type of angiogenesis are poorly known

The growth and development of new blood vessels from the pre-existing vascular bed is a highly controlled multistep process known as angiogenesis (Fig. 1). This process has well been studied in literatures [12–14]; it occurs in both natural growth and development as well as in the progression of diseases such as cancer. Two mechanisms are involved in angiogenesis: sprouting angiogenesis and intussusceptions [12, 15]. Previous studies suggest that hypoxia preferentially leads to sprouting angiogenesis, while hemodynamic factors induce intussusceptive angiogenesis. Although the detailed mechanisms of sprouting angiogenesis are more understood, the exact mechanisms that regulate intussusceptive angiogenesis are not fully understood. In general, there are two key cell types such as ECs and mural cells in vessels that regulate angiogenesis. Besides, different factors including, VEGF, angiopoietins, EGF, FGF, and TGF-α promote angiogenesis, however, there are also angiogenesis inhibitors like thrombospondin-1/2, angiostatin, interferons, collagen IV fragments, and endostatin [12–14]. Matrix metalloproteinases (MMPs), including MMP1 and MMP2, degrade the capillary wall by disbanding the basement membrane. Once degraded, a new branch point is formed within the wall of the existing vessel [16].

Sprouting angiogenesis is multistep, so inducing sprout and tip cell formation are the initial step for angiogenesis [12] (Fig. 1a). Sprouting is regulated by the balance between pro-angiogenic factors including VEGF and factors that induce quiescence like pericyte contact and VEGF inhibitor. Tip cells have a critical role for new vessels grow [17]. In conditions that favor angiogenesis, some ECs react to angiogenic factors like VEGF-A and sprout, while others fail to respond [17]. These cells called ‘’tip cells’’; therefore, VEGF-A authorizes ״tip cells״ for invasion and migration [17] (Fig. 1a). Selection of “tip cells” is organized by Notch family receptors and their transmembrane ligands DLL4 (Delta-like ligand 4) [18]. Expression of DLL4 and its Notch receptors are activated by the interaction of VEGF with endothelial cells [19]. Tip cells sprout is directed by VEGF gradients, which is mediated by the interaction of VEGF-VEGFR-2. These cells act as motile guidance cells that dynamically spread filopodia to discover attractive or revolting signals that are present in the environment [12, 18]. However, following the tip cells, there are endothelial stalk cells that have fewer filopodia but are highly proliferative and support adherent and tight junctions to promote the stability and formation of the budding vascular lumen [17, 20] (Fig. 1a). In this scenario, VEGF-VEGFR2 signaling orchestrates migration and proliferation of ECs, recruitment of pericytes, and tube formation [21]. Other factors may include abhorrent or attractive matrix cues and guidepost cells in the tissue environment. Transformation of sprouts into mature vessels consists of inhibition of ECs proliferation and migration of new capillaries, the stability of already existing new vascular tubes (fusion of the newly formed vessels with others), and enrollment of mural cells including pericytes and vascular smooth muscle cells [22, 23]. Different signaling pathways contribute to the endothelium/pericytes cross talk, of which three of the best known are angiopoietin-1 (ANG-1)/Tie2, transforming growth factor (TGF-β)/TGF-R, and ephrinB2/EphB4, promoting endothelial quiescence and new vessel stabilization [12, 22, 23].

Intussusception seems to bean energetically and metabolically process than sprouting angiogenesis owing to relatively low cell proliferation and prevention of the extracellular matrix degradation [24]. Hemodynamics forces are a major contributing factor for inducing intussusceptive angiogenesis, additionally, VEGF-A plays an essential role in shear stress-based splitting of capillaries [24, 25]. This process has already been defined in the chicken chorioallantoic membrane (CAM) model and also in skeletal muscle [24, 25]. As shown in Fig. 1b, it is characterized by the establishment of intraluminal tissue pillars, leading to the splitting of vessels [25]. The formation of intraluminal pillars continues through a multistep event that initiates with the protrusion of the opposite ECs membrane into the vascular lumen (Fig. 1b). In keeping, the fusion of the ECs protrusions forms the intraluminal pillars and the ECs links are restructured, so that a gap is shaped in the center of the pillar. Next, this pillar is occupied by supporting cells such as fibroblast and pericytes, which produce the extracellular matrix. Consequently, these events lead to split the vascular fragment into two isolated new vessels, branching vessel [24, 25] (Fig. 1b).

### Definition of EVs

**Fig. 2:**
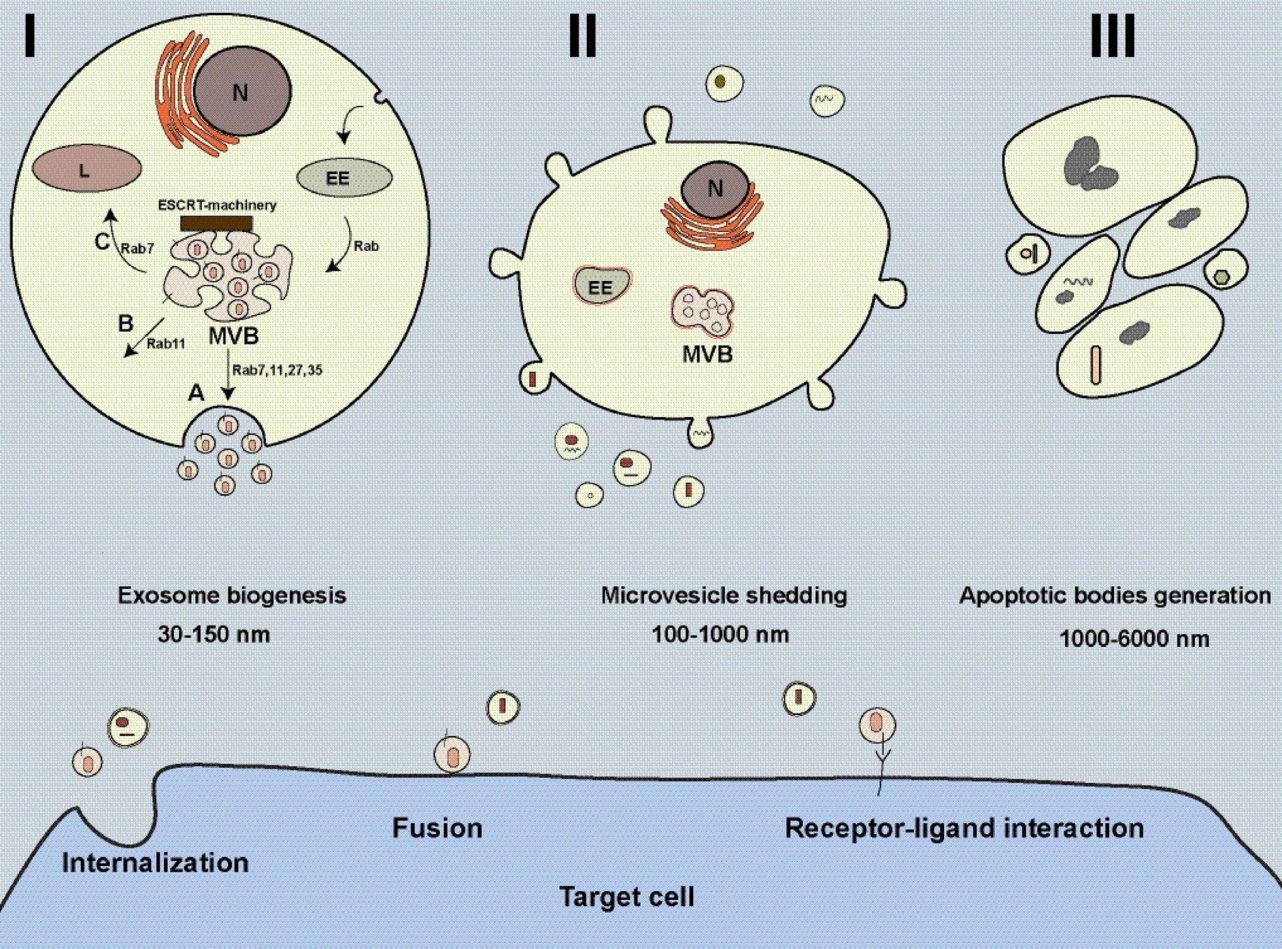
Extracellular vesicles biogenesis. Exosomes are originating from multivesicle bodies (MVBs) located in the cytoplasm (I). MVBs have three fates including secretion (A), back-fusion (B), and degradation (C). Microvesicles (MVs) which known as sheding vesicles are releasing from the plasma membrane of cells. Apoptotic bodies (ABs), the largest extracellular vesicles, generating from cells undergone apoptosis. Extracellular vesicles can deliver their cargo to target cells through three possible ways like endocytosis, receptor-ligand interaction, and direct fusion with the plasma membrane. *EE* early endosome, *L* lysosome, *N* nucleus

Extracellular vehicles (EVs), heterogeneous phospholipid bilayer spherical bags, are released from a variety of cell types. Exosomes and microvesicles and large vesicles are the most recognized subclasses of EVs, which have gained attention due to their pivotal roles in diseases [26] (Fig. 2). EVs as intracellular communication tools contribute to regulating neighboring cells function via delivering many kinds of materials such as nucleic acids, proteins, lipids, and carbohydrates [26]. These vesicles are present in almost bio-fluids including blood plasma, breast milk, urine, bile, cerebrospinal fluid (CSF), bronchoalveolar lavage fluid, saliva, peritoneum, and semen [7, 27]. Established in 2011, International Society for Extracellular Vesicles (ISEV) is a globally scientific organization that concentrates on the study of EVs including microvesicles (MVs), exosomes, oncosomes, and other membrane-bound vesicles that are produced by cells. According to the guidelines of ISEV, the terms apoptotic bodies (ABs), MVs, and exosomes have been traditionally used for the cataloging of the three main EVs subpopulations. This traditional classification is based on EVs origin, size, and specific markers. ABs are the largest EVs (1000–6000 nm) originating from apoptotic cells (Fig. 2). MVs, shedding vesicles, releasing directly from the cell membrane in both physiological and pathological conditions, represent 100–1000 nm with an irregular shape (Fig. 2). Exosomes the smallest subpopulation of EVs (30–150 nm) generating from endosomal compartments inside cells [28]. In the next section, we describe exosomes biogenesis and secretion.

### Exosomes biogenesis and trafficking

Exosomes are formed from the distinct endocytotic vesicles known as multivesicular bodies (MVBs) located at the cytoplasm [29] (Fig. 2). Inward budding of MVBs membrane generates intraluminal vesicles (ILVs) inside MVBs, which are secreted into the extracellular matrix as exosomes upon MVBs-the plasma membrane fusion [7]. Two pathways are involved in exosomes biogenesis, known as endosomal sorting complex required for transport (ESCRT) machinery‐dependent and ESCRT‐independent machinery [26, 29]. ESCRT-machinery located on MVBs membrane and composed of four complexes known as ESCRT-0, ESCRT-I, ESCRT-II, ESCRT-III and accessory proteins that contribute to separate and sort ubiquitylated proteins into nascent ILVs and abscission of ILVs into the MVBs lumen using ATP molecules [26, 29]. Through the ESCRT‐independent machinery, molecules other than ESCRT-dependent machinery contribute to exosomes biogenesis, for example, ceramides are the waxy lipids that mediate exosomes biogenesis. A ceramide is composed of sphingosine and a fatty acid that can induce MVB membrane curvature and the formation of ILVs into MVB’s lumen [30, 31]. Other molecules such as syndecan-syntenin-ALIX complex [32, 33], VCAM-1 and α4 integrin [34, 35], phosphatidic acid (PA) [36], and tetraspanin like CD63 [37], CD9, and CD82 [38] are involved in exosomes biogenesis. According to literature, MVBs have three possible intracellular fates including secretion, degradation, and back-fusion (Fig. 2). In the secretion pathway, MVBs fuse with the plasma membrane and ILVs are released into the extracellular matrix as exosomes. In the degradation pathway, MVBs fuse with lysosomes, and then their content is hydrolyzed, while in back-fusion pathway MVBs combine with the plasma membrane and decorate it with receptors and other molecules.

Different Rab proteins such as Rab7, Rab8, Rab11, Rab27, and Rab35 facilitate intracellular trafficking of MVBs [39–43] (Fig. 2). SNARE proteins [soluble N-ethylmaleimide-sensitive fusion attachment protein (SNAP) receptors] mediate the fusion of MVBs with the plasma membrane [44]. Although exosomes from different cells contain various molecules, but they contain the conventional markers like CD81, CD82, CD63, CD9, TSG101, and also ALIX [45].

As shown in Fig. 2, three pathways have been suggested for exosomes that affect target cells as: endocytosis, receptor/ligand interaction, and direct fusion with the plasma membrane of target cells [46, 47]. Exosomes can reach target cells through different endocytosis pathways including phagocytosis, pinocytosis, and receptor-mediated endocytosis [46, 47]. Exosomes may dock at the plasma membrane of the target cell and activate/inhibit intracellular signaling by ligand-receptor interaction. Direct fusion is another way by which exosomal membrane fuse directly with the target cell membrane and exosomes content discharge into the cytoplasm of the target cell. Understanding the detailed mechanisms behind exosome delivery pathway is worthy for designing exosome-based therapies.

### Exosomes cargo

Exosomes contain different types of biological molecules transferred from source cells to target cells [48]. Analysis of exosomes cargo has received much attention in the past decade because identifying exosomes cargo improve our knowledge about detailed mechanisms involved in formation, loading, and also key functions of exosomes in different conditions; and further, provide us a new avenue to use them as a biomarker and therapeutic approaches for the treatment of various diseases [49]. Several databases have been established to collect and present exosomes cargo from different sources. For example, Exocarta (http://www.exocarta.org) has presented about 563 proteins, 4764 miRNAs, 1,639 mRNAs, and 194 lipids of exosomes from various organisms [50]. In addition, Vesiclepedia (http://www.microvesicles.org) has presented about 1254 EVs-related studies and classified nearly 38,146 different RNA molecules, 349,988 proteins, and 639 lipids [51]. In 2019, a database (http://bioinfo.life.hust.edu.cn) has been established to analyze small RNA sequencing of different EVs from various sources [52]. Cell condition can affect exosomes cargo and alteration in them may be used as a biomarker of such diseases [53].

### Protein cargo

Protemome profiling of exosomes have characterized different types of protein within exosomes and also on exosomes membrane [54–56]. Different exosomes represent distinct cargo as their source cells have an individual profile; however, as mentioned above, exosomes from different sources contain common markers. Exosomes harbor membrane molecules including integrins, adhesion molecule 1 (ICAM-1), cytoskeleton components (annexins, actin, and tubulin), component from endocytosis pathway like TSG101 and Alix proteins; exosome loading molecules such as CD63, CD81, and CD9, and intracellular trafficking molecules like various Rab and SNAREs proteins, and also many other signaling proteins [28, 57]. As mentioned previously, several mechanisms have been involved in the specific sorting of proteins into exosomes, such as ESCRT, tetraspanins, and lipid-based mechanisms. Additionally, exosomes contain common lipids for example ceramides, phosphatidylethanolamine, phosphatidylserine, diacylglyceride, bisphospatidic acid, sphingomyelin, and cholesterol [58].

### Nucleic acid cargo

In addition to proteins, exosomes also are full of different types of RNAs that can be delivered into recipient cells. Using RNA sequencing analysis, Huang et al. confirmed that miRNAs were the most abundant in exosomes purified from the human plasma, making up over 76.20% of all mappable reads and 42.32% of all raw reads [59]. Other RNA species such as long non-coding RNA (3.36%), ribosomal RNA (9.16%), transfer RNA (1.24%), piwi-interacting RNA (1.31%), small nuclear RNA (0.18%), and small nucleolar RNA (0.01%) have been characterized in exosomes. Once miRNAs are loaded into exosomes, they can transfer between cells, resulting in an intercellular trafficking network, which, in turn, induces functional and phenotypic changes in recipient cells [60]. Different kinds of miRNAs are present within various exosomes, for example, miRNAs such as miRNA-1, miRNA-21, miRNA-29a, miRNA-214, miRNA-320, and miRNA-126 mediate regulation of angiogenesis, exocytosis, metastasis, hematopoiesis, and tumorigenesis [61]. In addition, a growing body of evidence showed that long RNAs like long non-coding RNAs (lncRNAs) and circular RNAs (circRNAs) are loaded into exosomes, and they participate in a variety of biological processes such as cancer [62]. For instance, lncRNA TUC339 was the most highly expressed one in exosomes from human hepatocellular cancer, which is involved in tumor cell growth and adhesion [63]. It seems that the exosomal RNAs loading process is a regulated and complex mechanism [64]. Cells use distinct mechanisms for sorting specific nucleic acids into the exosomes [65]. DNA strands have recently been revealed to be transferred by exosomes. Exosomes derived from sera of Pheochromocytomas and paragangliomas patients, heritable endocrine tumors, contain DNA strands that would be used as biomarker of these tumors [66]. Furthermore, the presence of DNA strands within exosomes has been suggested to be associated with processes such as cell senescence and inflammation [67]. As we know, DNA is mostly restricted to the nucleus and does not usually interact with the cytoplasmic the exosomal secretory pathway for secretion [67]. Although micronuclei as cytoplasmic structures may likely participate in loading of DNA strands into exosomes, the underlying mechanisms remain mysterious [68].

### Tumor cell derived-exosomes and angiogenesis

**Fig. 3:**
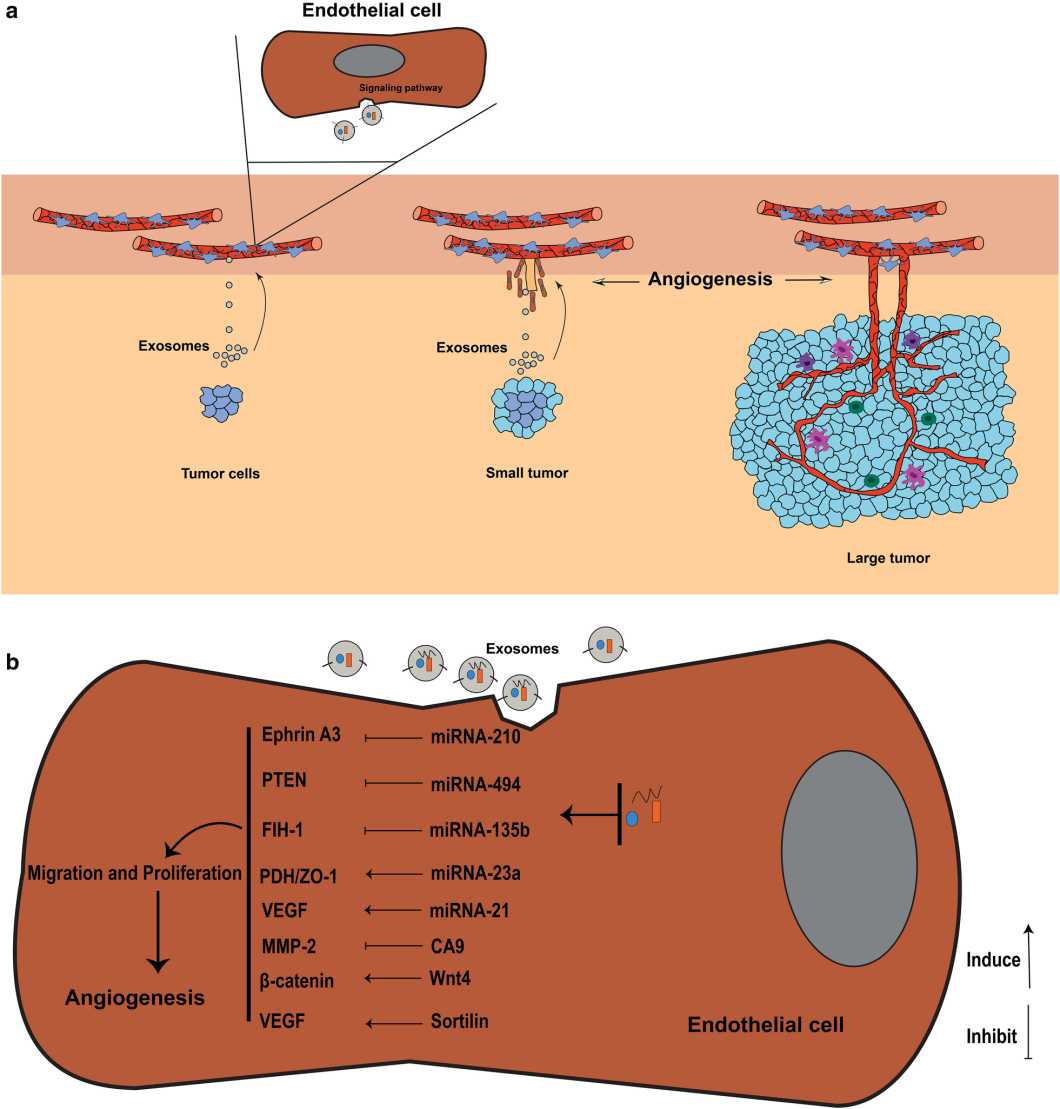
The key role of exosomes from tumor cells in angiogenesis (**a**). Exosomes derived from tumor cells contain various proteins and RNAs that promote angiogenesis through different signaling pathways upon delivering to endothelial cells (**b**)

Tumor derived-exosomes are essential factors for the formation of new vessels at the early stage of tumor progression (Fig. 3). Exosome purified from primary human malignant mesothelioma (MM) can induce migration, vascular remodeling, and angiogenesis in a MM model [2]. Proteomic analysis showed that these exosomes contain oncogenic cargo inducing cell migration and tube formation molecules [44]. Murine multiple myeloma derived-exosomes have been shown to induce the formation of the metastatic niche in bone marrow and promote angiogenesis in vivo [69]. Exosomes derived from Glioblastoma multiforme (GBM), a vascularized and aggressive type of brain cancer [70], are full of angiogenic proteins that promote angiogenesis in vitro and in vivo models [71, 72]. These exosomes support ECs proliferation and tubulogenesis. Similar results have been reported by Skog et al, who found that exosomes from GBM cells contain mRNAs belonging to ontologies like angiogenesis, and increased tubulogenesis of human brain ECs in vitro [73]. In other solid tumors, for example, nasopharyngeal carcinoma, proteomic analysis showed that pro-angiogenic proteins were increased, while antiangiogenic proteins were decreased [74]. These exosomes induced migration and tubulogenesis of ECs. Similarly, exosomes derived from different breast cancer cell lines and pancreatic carcinoma cells are angiogenic and induce angiogenesis [75–77]. Exosomes released from hypoxic lung cancer cells transfer miRNA-23a to ECs that promotes angiogenesis through targeting tight junction protein ZO-1 and prolyl hydroxylase and increasing vascular permeability [78]. Besides solid tumors, exosomes from chronic myelogenous leukemia (CML) cells have been shown to promote angiogenesis via direct interaction with ECs [79, 80].

### Exosomes uptake by ECs

Exosomes can interact with target cells such as ECs and immune cells to facilitate angiogenesis. Exosomes-mediated reprogramming in the recipient cells is depended on the exosomes uptake routes. Exosomes can affect T cells via the direct receptor-ligand interaction, however in the ECs case, exosomes use internalization pathway to affect ECs [71, 80, 81]. PKH26-labeled exosomes have been shown to deliver their cargo into the cytoplasm of ECs after 4 h of co-culturing ECs cells with PKH26-labeled exosomes [80, 82]. In ECs, the endocytosis pathway may be the main route for uptaking exosomes [83]. After entry exosomes, they are directed to the perinuclear zone and traffic to cortex and enter into the actin filaments richen area forming pseudopods during tubulogenesis [80]. Interestingly, exosomes in these points are found in clusters and after cellular remodeling, these exosomes may move to other neighboring cells by nanotubular structures detectable by confocal microscopy. These observations may support the idea that ECs during tubulogenesis communicate with other ECs and other surrounding cells within the tubule network [80, 82]. Even though the uptake ways of tumor derived-exosomes by ECs is the mostly studied, other mechanisms may be involved, particularly, the receptor-ligand interaction [84].

### Mechanisms involved in exosomes-induced angiogenesis

Once internalized into recipient cells, exosomes cargo can regulate fate, function, and phenotype of recipient cells [71, 85]. Exosomes docking on cell surface may activate/inhibit the signaling pathway in ECs through receptor-ligand interaction [86]. Therefore, exosomes can engage different signaling pathways of recipient cells to affect recipient cells function [87]. The exact signaling pathways behind angiogenesis driven by exosomes are poorly known. The pivotal roles of protein cargo of the cancer derived-exosomes in cancer progression and angiogenesis have been documented [88, 89]. The proteomic content, even angiogenic profile of exosomes from different tumor cells widely differ in various tumor cells (Table 1). However, these differences may arise from the bias of researchers in targeting proteins of interest. Analysis of exosomes from GMB cells showed that these exosomes are enriched with pro-angiogenic factors including VEGF, angiogenin, TGFβ, IL-8, IL-6, MMP2, MMP9, TIMP-1, TIMP-2, and CXCR4 chemokine receptor [73, 90, 91]. MM-derived exosomes abundantly contain bFGF, VEGF, HGF, Serpin E1, and MMP-9 [69]. Exosomes from nasopharyngeal carcinoma cells contain a high level of pro-angiogenic proteins including CD44 isoform 5 (CD44v5), ICAM-1, and MMP13, while contain a low level of antiangiogenic protein, thrombospondin-1 [74, 92].

**Table 1:** Angiogenic cargo of tumor cell-derived exosomes

Source cells	Angiognic cargo
Breast cancer	Annexin II, Heparanase, TGFβ, miR-126a
Bladder cancer	EDIL-3
Colorectal cancer	Plexin B2, Tetraspanin-8
Glioblastoma	Angiogenin, CXCR4, FGFa, IL-6, IL-8, MMP2, MMP9, TGFβ, TIMP-1, TIMP-2, VEGF
Lung cancer	Sortilin, miRNA-21, miRNA-23a, miRNA-210
Glioma	IGFBP1, IGFBP3, IGFBP5, IL-8, LOXL2, VEGF
Ovarian cancer	miRNA-21
Leukemia	Heparanase
Nasopharyngeal cancer	CD44v5, HAX-1, ICAM-1, MMP13
Malignant melanoma	VEGF, MMP2, IL-6
Multiple myeloma	Angiogenin, Heparanase, HGF, MMP9, Serpin E1, Serpin F1, VEGF

You et al. found that these exosomes contain HAX-1 protein that induces migration ability and angiogenesis in ECs [92]. Analyzing exosomes from the colorectal carcinoma ascites showed that these exosomes carry angiogenic proteins like Plexin B2 and tetraspanin-8 [93]. Melanoma cancer released-exosomes bear VEGF, IL-6, and MMP2 [94]. Exosomes from lung adenocarcinoma are enriched with sortilin, which increases the expression of angiogenic genes including IL-8, VEGF, endothelin-1, thrombospondin-1, and uPA in ECs [95]. Breast cancer derived-exosomes transfer proangiogenic Annexin II to ECs and induce angiogenesis via the tPA-dependent manner in vitro and in vivo [76]. Beckham et al. declared that exosomes from bladder cancer patients contain EDIL-3 proteins that facilitate migration and angiogenesis [96]. Heparanase, an enzyme involved in exosomes biogenesis and loading, is present in tumor cell derived-exosomes and contributes to migration and tube formation of ECs [97, 98]. Prostate cancer released exosomes contain TGF-β1 proteins that mediate differentiation of fibroblast into myofibroblast, promoting angiogenesis in vitro [99]. Exosomes produced by pancreatic adenocarcinoma have a high level of Tspan8 that promote proliferation, migration, and sprouting in ECs. Furthermore, these exosomes mediate maturation of endothelial progenitor cells [100]. Tumor derived-exosome can induce epithelial-mesenchymal transition (EMT) in different cancer cells. EMT cells produce exosomes with angiogenic Rac-1 and PAK-2 proteins that induce angiogenesis in ECs [101]. In GBM cells, Zeng et al. showed that EMT cells derived-exosomes induced cell migration, invasion, and angiogenesis [96]. Nucleic acids content of tumor-derived exosomes mediate angiogenesis in ECs upon exosome internalization. For example, colorectal cancer cells release exosomes transferring proliferation-related mRNAs such as RAD21, CDK8, and ERH to ECs and increase proliferation of ECs and subsequently support angiogenesis [102]. Lang et al. found that GBM-derived exosomes contain lncRNA POU3F3 that promote angiogenesis in ECs [71]. In addition, in another study, it was demonstrated that these exosomes transfer lncRNA-CCAT2 to ECs, which subsequently inhibits apoptosis and enhances angiogenesis [71]. Enriched in exosomes, miRNAs can deliver into target cell cytoplasm and control different mRNAs expression and cell function of target cell [103]. Obviously, exosomal miRNAs fascinated attentions have key roles in increasing the adversarial effects of tumors [104]. For example, in human nasopharynx cancer, tumor-derived exosomes actively transfer miRNAs including miRNA-106a-5p, miRNA-891a, miRNA-24-3p, and miRNA-20a-5p that promote cell proliferation and survival through suppression of MARK1 protein signaling pathway [105]. miRNAs cargo of tumor-derived exosomes are also involved in angiogenesis through regulating ECs function and morphology [106, 107]. Exosomes protect miRNAs from enzymatic degradation, thus increases the stability of exosomal miRNAs compared to circulating ones. In Table 2 a list of angiogenic exosomal miRNAs is presented. Lung cancer cells release exosomes enriched with miRNA-21. This miRNA is an oncogenic and angiogenic molecule that enhances expression and secretion of VEGF, inducing angiogenesis in ECs [108]. Other miRNAs such as miRNA23a and miRNA-210 which are present in exosomes of lung cancer cells and are implicated in inducing angiogenesis in ECs [78, 109]. Besides, exosomal miRNA-192 has been shown to inhibit angiogenesis [110]. Umezu et al. demonstrated that hypoxia-resistant multiple myeloma (HR-MM) cells release exosomes containing miR-135b that enhance angiogenesis in ECs through targeting HIF-1 [111]. Lung cancer cells secrete exosomes enriched with miR-23a, which facilitate the angiogenesis by targeting tight junction protein ZO-1 and prolyl hydroxylase [78]. Mao et al. found that hypoxia increased miR-494 loading into exosomes of non-small cell lung cancer (NSCLC) through the HIF-1α-mediated mechanism. In keeping, they showed that these exosomes down-regulated PTEN and activated Akt/eNOS pathway in ECs and consequently promoted angiogenesis [112]. In addition, miR-210 cargo of exosomes purified from leukemia cells induced the tubulogenesis in human endothelial cells [113]. The possible mechanisms that tumor-derived exosome cargos promote angiogenesis have been presented in Fig. 3b. The biomarker potential of exosomal miRNAs has frequently been reviewed in literature [114, 115]. As miRNAs bearing exosomes can be distributed to bio-fluids, therefore, liquid-biopsy from urine, plasma, and CSF is a non-invasive method for obtain exact information about tumor environment/status [116]. For example, it was demonstrated that miRNAs such as miRNA-205, miRNA-214, miRNA-141, miRNA-203, miRNA-200 a,-b,-c, and miRNA-21 are present in exosomes isolated from patients suffering from ovarian tumors and they could be serve as biomarkers [117].

**Table 2:** Hypoxia-induced angiogenic cargo of tumor cell-derived exosomes

Source cells	Angeogenic factors
Breast cancer	TGFβ
Leukemia	miR-18b, -20a, -24, -106b, -130b, -185, -210, 224, -379, -652
Glioma	IGFBP1, IGFBP3, IGFBP5, IL-8, LOXL2, VEGF
Multiple myeloma	miR-135b, -200c, -210, -223, -328, -335, -425
Lung cancer	miR-23a

### Exosomes from hypoxic cells

Hypoxia plays a critical role in inducing tumor angiogenesis. In tumors with a high level of growth and metabolism rate, oxygen deficiency, thus hypoxia contributes to induce angiogenesis via hypoxia-inducible transcription factors [118]. Under hypoxic condition, cancer cells release more exosomes, which represent proangiogenic properties [9]. In addition, researchers have declared that exosomes from hypoxic tumor cells are enriched with exosomal markers such as CD81, CD63, and HSP-70 [78, 113, 119]. Normoxic condition is another factor that affects components of exosomes. Hypoxic cells release exosomes differing from those derived from normoxic cells. For example, exosomes from hypoxic GBM cells contain higher levels of IGFBP3, IGFBP5, and LOXL2 than those from the same cells exposed to normoxic conditions [120]. In support, Kucharzewska and co-workers showed that these exosomes significantly promoted proliferation, migration, and tubulogenesis of recipient ECs as compared to exosomes from normoxic cells [72]. They confirmed that hypoxic exosomes have high mRNA and protein levels of IL-8 and IGFBP3 that induce proliferation and migration of pericytes, the angiogenic cells in vitro [72]. However, some researchers indicate exosomes from both hypoxic and normoxic cells represent the same physical features and by the same way deliver their cargo to ECs [111, 113]. Similarly, under hypoxic culture condition, exosome from lung and leukemia cancer cells increased permeability of ECs and angiogenesis [78, 113]. Hypoxia also alters miRNA cargo of exosomes from different cancer cells including lung cancer cells and MM cells [78]. Huang et al. found that exosomes released from hypoxic colorectal tumor cells induced angiogenesis via HIF-1/Wnt4/β-catenin signaling pathway in ECs [111]. These facts show that hypoxic condition which is frequently observed in the tumor environment plays a key role in tumor angiogenesis, therefore in tumorigenesis; thus it seems that it is a promising approach to design new treatment strategies against hypoxia-induced angiogenesis.

### Targeting exosome-induced angiogenesis

Understanding the molecular mechanisms behind exosome-induced angiogenesis is the key factor for the progression of a new approach for cancer therapy. It seems likely that new therapeutic strategies which target exosome biogenesis and/or exosome-induced angiogenesis can reduce tumorigenesis. Most types of tumors are vascularized and produce exosomes, thus it would be major progression to discover the underlying mechanisms involved in angiogenesis driven by tumor-derived exosomes and to identify the ways for inhibiting angiogenesis and thus, improve the current therapies outcome. Corrado et al. reported that Carboxyamidotriazole orotate (CTO) decreased angiogenesis ability of imatinib-resistance CML cells [121]. CTO targets the expression of IL-8 and cellular adhesion of ECs, which promoted by tumor exosomes. Thus, CTO inhibits the action of these exosomes on ECS-CML interaction and migration of ECs, suppression exosome-induced angiogenesis [121]. Treatment of CML cells with curcumin altered exosomes cargo. In this regard, curcumin increased miRNA-21 and antiangiogenic proteins sorting into exosomes but decreased sorting of proangiogenic proteins into exosomes. Consequently, ECs lost their function upon uptake these exosomes in vitro [122]. In support, Docosahexaenoic acid (DHA) which used as an adjuvant to breast cancer therapy has been shown to target exosomes loading and biogenesis. DHA treatment increased the level of miRNA-23b, miRNA-320b, and miRNA-27b in exosomes. Furthermore, these exosomes are internalized by ECs and suppressed tubulogenesis in ECs without affecting VEGF expression [123].

Pharmacological inhibitors which are capable of targeting exosome biogenesis, loading, and secretion may be a potential agent for inhibiting angiogenesis [124]. For example, GW4869 and Manumycin A have been reported to block exosomes formation from MVB. Other compound such as Calpeptin, Y27632, and Imioramine can inhibit MVs formation [124]. Datta and colleagues showed that manumycin A inhibited exosome biogenesis in prostate cancer cell lines (C4-2B, PC3 and 22Rv1) [125]. Indeed, Manumycin A inhibits Ras activity, a small GTPase involved in exosome biogenesis [126]. It has been reported that chemicals, compounds, and peptides can inhibit EVs uptake by target cells, which may suppress EVs function on target cells [47]. However, despite progress in EVs biology, the detailed mechanisms behind their generation and function are still elusive. Furthermore, the main concern is that these compounds do not specifically target tumor cells and may show side effect on other cells, suppressing promising EVs. Important efforts would still necessary to examine their impact on EVs secretion from normal cells. Approaches to preferentially distribute them to tumor cells may be vital. Certainly, the drugs that are previously approved for use in humans, due to some indications, might have a more straightforward way to usefulness than those are compounds that have not ever been established as therapeutics.

### Cancer stem cell derived-exosomes and tumor angiogenesis

Cancer Stem Cells (CSCs), a small subpopulation of self-renewal cells within tumors, give rise to heterogeneous tumor cells populations that make up tumor. CSCs produce angiogenic exosomes containing stem cell markers including CD44, CD133, CD90, and CD105 [127, 128]. Grange et al. found that exosomes from human renal cancer stem cells express CD105 marker, which induced angiogenesis and facilitate the metastatic niche formation [127]. Conigliaro et al. declared that CD90 positive exosomes from liver cancer cells increased tube formation and cellular adhesion in ECs. Further scrutiny showed that these exosomes significantly up-regulated the expression of VEGF and its receptor [82]. Besides, miRNA cargo of exosomes from human prostate cancer cells is different from those of bulk cells. These findings indicate exosomes from CSCs represent distinct cargo, and therefore CSCs stand for a promising target for therapies.

### Mesenchymal stem cells derived-exosomes and tumor angiogenesis

Mesenchymal stem cells (MSCs), self-renewal cells, can differentiate into various lineages such as osteoblasts, fibroblasts, adipoblasts, chondroblasts, pericytes, and even other cell types [129]. They usually used as a source of cell therapy owing to their profound regenerative capability and immunosuppressive effects [130]. One of the fundamental mechanisms of MSCs usefulness appears to rise from their paracrine activity. Exosomes from MSCs orchestrate the main mechanisms of action of MSCs after transplanting into target sites [131]. The critical role of MSCs-exosomes in tumor proliferation, invasion, and also angiogenesis is still controversial. Some laboratories declared that these exosomes support tumorigenesis, however, others found that they suppress tumor tumorigenesis [132], thus, MSCs‐exosomes represent a dual effect on tumor angiogenesis. In the case of antiangiogenic effects, it was demonstrated that exosomes from mouse bone marrow (BM) MSCs inhibited angiogenesis in breast cancer cells via suppressing VEGF expression [133]. The authors declared that miRNA-16 within these exosomes down-regulates VEGF. Pakravan et al. demonstrated that exosomes from BM-MSCs contain miRNA-100 that declined VEGF expression in breast cancer cells and suppressed angiogenesis in vitro by modulation the HIF‐1α/mTOR signaling [134]. miR‐100 is antitumor miRNA and down-regulated in different cancer cells. Thus, exosomal transfer of miRNA-100 compensates low level of miRNA-100 in tumor cells and participates to inhibit tumorigenesis. Further, authors found that conditioned media from MSCs exosome-treated breast cancer cells inhibited migration and proliferation of ECs [134]. Recently, Rosenberger et al. found that exosomes from menstrual MSCs had potential to inhibit angiogenesis in ECs via increasing apoptosis and inhibiting VEGF secretion [135]. Similarly, exosomes from menstrual MSCs blocked angiogenesis in prostate PC3 tumor cells via inhibition of VEGF secretion, NF-κB activity, and producing reactive oxygen species (ROS) [136].

In contrast, exosomes from MSCs increased tumor growth and angiogenesis. For example, Human BM-MSCs have been shown to increase angiogenic molecules in the gastric tumor in vivo. Co-implantation of SGC‐7901 cells with MSCs exosomes up-regulated the transcript level of α‐SMA, VEGF, MDM2, and CXCR4; and protein levels of VEGF, Bcl‐2 phosphorylated ERK1/2, and CXCR4 [137].

### Mesenchymal stem cells derived-exosomes as drug-carriers

Anticancer drugs have several disadvantages including side effects on normal tissues, solubility, short half‐life, and limitation in the passing through the physiological barriers, hence, nanocarriers have been advanced to overcome these limitations [138, 139]. Recently, nanocarriers such as liposome, ligand‐conjugated nanoparticles, and magnetic nanoparticles have been examined for delivering therapeutic agents to cancer cells. However, these nanoparticles sometimes have limitations due to their synthetic structures and non-targeting effects on tissues [140–142]. Compared to synthetic carriers, EVs are safe, cell-origin, and natural carriers that show long half-time and non-immunogenic properties for drug delivery systems. They contain various proteins and nucleic acids, which can be modified by available techniques [143]. Besides, based on the origin of tissue/cell, EVs can home into their origin sites, which make them ideal and specific carrier for targeting tumor cells [144]. Overall, two strategies are used to design the exosome-based nanocarriers from cells and exosomes as I: direct engineering process and II: indirect engineering process.

In direct engineering process, exosomes purified from the optional cells directly engineered with exogenous therapeutic agents like synthetic compounds, drugs, and biomolecules. In indirect engineering process, source cell (MSCs/tumor cells) are genetically modified for producing optional exosomes or are incubated with the therapeutic drugs to load drugs into exosomes [145]. These exosomes now are carriers for therapeutic agents and known as exosome-based nanocarriers, which are capable of delivering drugs to target cells. In this regard, provide a high amount of exosomes at the same time being safe and non-immunogenic is the hallmark of exosome-based nanocarriers for delivering therapeutic agents. MSCs produce abundantly nonimmunogenic, beneficial, and safe EVs among other cells [146]. In addition, MSC‐EVs do not show some limitation such as malignant transformation, genetic variability, rejection, and cytotoxicity [147]. MSCs‐EVs play key roles in improving cardiovascular disease, liver disease, acute kidney injury, lung disease, and cutaneous wound healing [145, 148]. These facts support an idea that exosomes from MSCs may serve as the beneficial vehicle for cancer therapies.

### Clinical trials

**Fig. 4:**
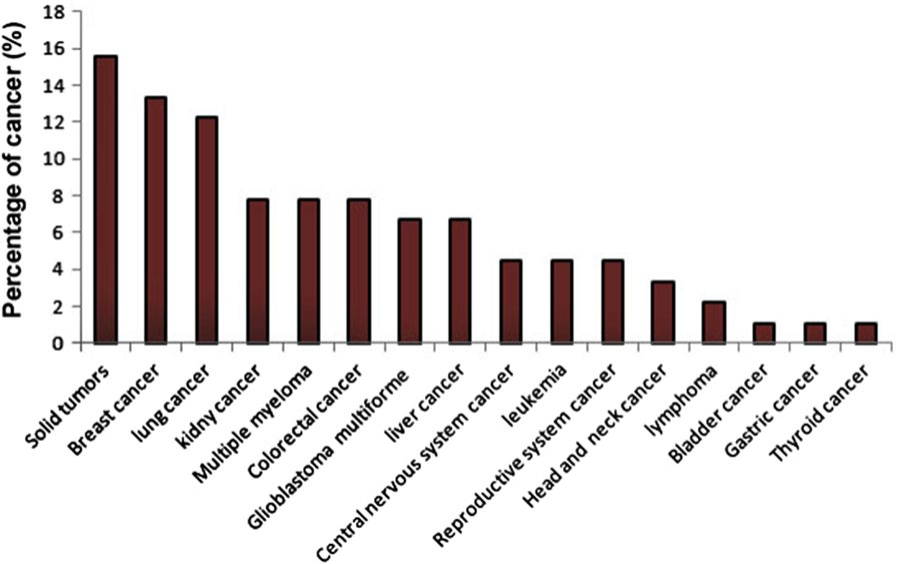
A diagram representing percentage of clinical trials related on angiogenesis in different cancers by 10 April 2020

Consistent with recent development in molecular mechanisms of tumor angiogenesis, clinical trials are becoming more common. By 10 April 2020, the National Institutes of Health at (Clinicaltrials.gov) recorded 90 clinical trials related to tumor angiogenesis in different cancers. A search through the records showed that the majority of clinical trials are identical to studies involving different solid tumors (15.55%) (Fig. 4). In addition, 13.33% and 12.22% clinical trials belong to breast and lung cancer respectively. All of these studies confirmed the fact that angiogenesis is a useful tool for cancer treatment in a clinical situation.

### Perspective

**Fig. 5:**
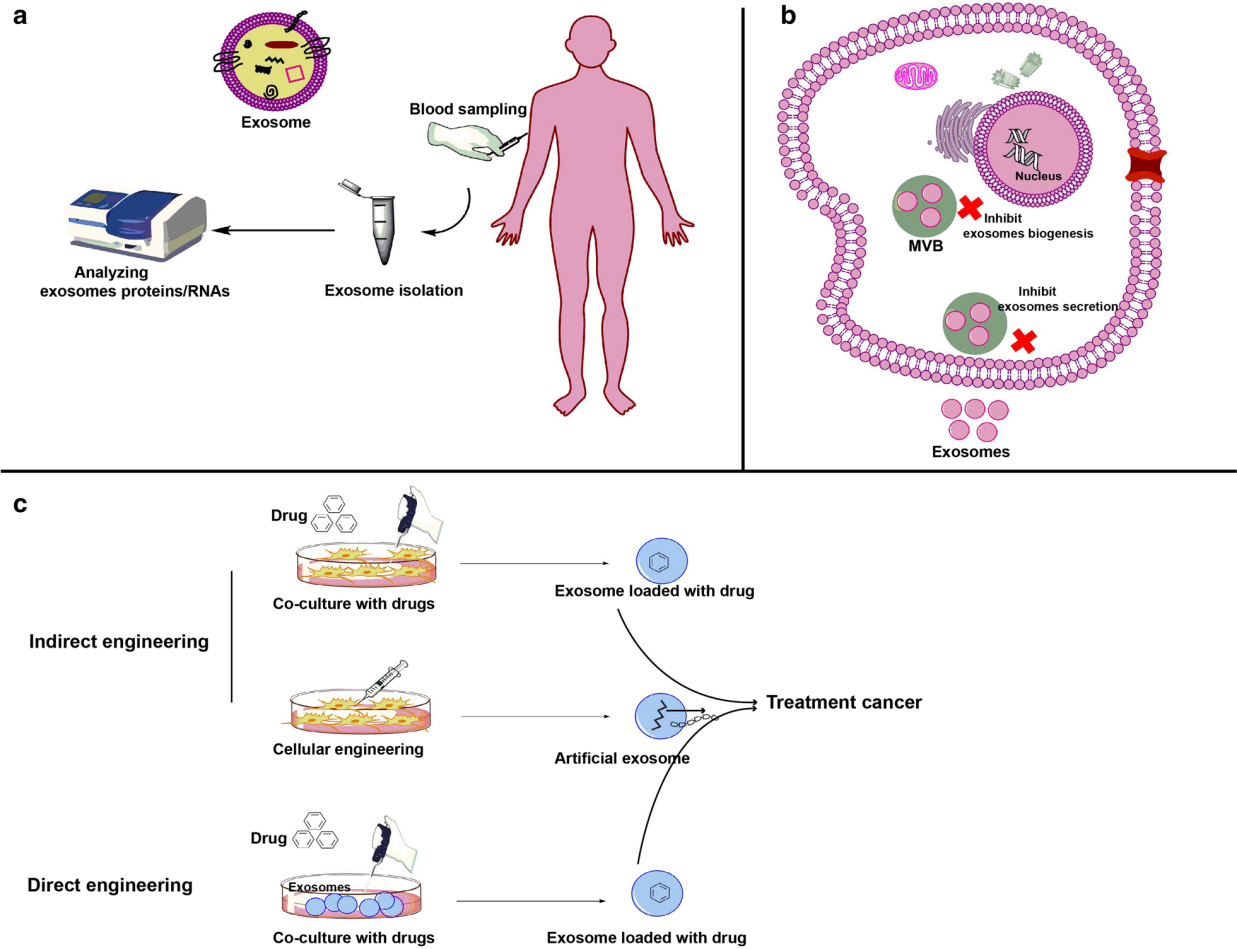
Possible therapeutic implications of exosomes. Exosomes may serve as biomarker for cancer diagnosis, staging, response to therapy and prognosis (**a**). Inhibiting of exosomes biogenesis and secretion from tumor cells have been suggested to reduce tumorigenesis (**b**). Exosome can be used as a drug delivery system. In this regard, source cells may be co-cultured with a drug to obtain exosomes containing drug or source cells may be genetically engineered to produce artificial exosomes. In addition, drugs are incubated with isolated exosome to load drugs into exosomes (**c**). Until now, exosome-based drug delivery system have been examined in vivo and in vitro

Cancer growth and metastasis are depended to angiogenesis, which is structured by a complex interaction between cells, molecular pathways, and soluble factors such as exosomes. The essential role of cancer cell derived-exosomes in angiogenesis has been recently described. These exosomes were found to be effective inducers of angiogenesis in vitro and in vivo through functional reprogramming and phenotypic modulation of ECs and other cells resident in the tumor microenvironment [71, 72]. Tumor cell derived–exosomes contain pro-angiogenic signaling molecules like proteins and RNAs. The formation of new vessels, which arises at an early step of the tumor growth, has been depended to the levels of exosomes from tumor cells. Cancer cells abundantly produce exosomes, and plasma of patients with cancer is enriched in tumor cell derived-exosomes. As the molecular content (such as miRNAs and proteins) of these exosomes recapitulate contents of the parent cell, they appear as possible non-invasive biomarkers of tumor progression and tumor angiogenesis [149] (Fig. 5a). This is hopeful for the design of a liquid biopsy model, which would allow for measurement of the tumor angiogenic profile in real time and repetitively. Application of these exosomes as biomarkers in following up of cancer progression or responses against antiangiogenic therapies enables to significantly improve patient management and also drug selection. They could develop as a tool for patient-specific diagnosis and server as personalized anti-angiogenic therapy. Thus tumor cell derived-exosomes may serve as future biomarkers of cancer diagnosis, staging, response to therapy and prognosis. Furthermore, upcoming efforts should focus on silencing or eliminating exosomes that selectively encourage malignant, but not benevolent, angiogenesis, thus adding novel treatment opportunities to current anti-angiogenic therapies [124] (Fig. 5b). An interesting approach has been suggested by Marleau and colleagues based on the effective elimination of circulatory exosomes by extracorporeal hemofiltration associated with affinity agents like exosome-trapping antibodies and lectins. This platform was proposed to capture and trap particles < 200 nm from the whole circulatory system [150]. However, this platform traps non-tumoral exosomes, which have normal physiological roles. Recent discoveries have revealed that it is possible to inhibit the biogenesis and release of exosomes from different cells [151]. Some researchers have endeavored to investigate exosome-inhibitors as research tools for exploring the exosomes kinetic; however, others assessed the inhibitory potential of such compounds in various disease models such as cancer [124, 151]. Most of experiments were done in preclinical, therefore, clinical trials are essential for validation and confirmation. However, the main concern remains about non-targeting effects of exosome-inhibitors (drug/compound) on exosomes biogenesis of healthy cells. For example, for cancer, it seems likely that substantial efforts would still be essential to study their effects on exosomes release from both healthy and tumor cells as well as to design methods to selectively deliver inhibitors to tumor cells. Exosome-therapy may be a promising tool for inhibition tumor. Select a popper sour cell to obtain exosomes for suppressing angiogenesis is a gold standard. Despite the promising function of MSCs-derived exosomes in regenerative medicine [152], as mentioned above, MSCs-exosomes exhibit both pro and anti-angiogenic properties, therefore the exact effect of their exosomes on tumor angiogenesis remain elusive. Another interesting approach that exosomes can be used as a therapeutic agent is the drug delivery potential of them [153–155] (Fig. 5c). Exosomes can serve as exosome-based nanocarriers that deliver therapeutic agent to target cells. Exosomes from a safe source such as MSCs may be loaded with anticancer/antiangiogenic compounds or genetically engineered for targeting tumor cells, suggesting the exosome-based nanocarriers for treatment of cancers in drug-delivery system. The advent of safe nano-carriers with high efficiency is the core goal of nano-medicine. Thus, the development of exosomes-based nanocarriers has opened a hopeful opportunity for the delivery of therapeutic agents. However, the majority of studies performed in vitro and animal models, therefore, the safety, specificity, and proficiency of this method in clinical trials remains still more mysterious. Our knowledge of EVs/exosomes biogenesis, loading, and function are still limited, therefore, to implement many of the ideas mentioned above, further studies of EVs (especially exosomes) from tumor cells are required.

## Conclusion

Tumor cell derived-exosomes have been shown to play a pivotal role in tumor angiogenesis, promoting tumor growth and metastasis. These exosomes contain various types of angiogenesis-related nucleic acids and proteins that trigger functional and phenotypic changes in ECs and support the vessel formation and growth. Tumor derived-exosomes may serve a potential biomarker for diagnosis of cancer. In addition, suppression of exosomes biogenesis from tumor cells may prevent tumor angiogenesis. However, future studies on the current topic are therefore required to elucidate the biological role of tumor cell derived-exosomes in tumor angiogenesis and to determine the clinical application of targeting these exosomes for preventing angiogenesis. Besides, the key role of exosomes from MSCs in tumor angiogenesis is still controversial. Due to favorable features, MSCs-derived exosomes may be useful in exosome-based nanocarriers for drug delivery. This is a vital issue for future research in exosomes-based cancer therapy.

## Data Availability

The primary data for this study is available from the authors on direct request.

## Abbreviations

ABs: Apoptotic bodies
BM: Bone marrow
CD44v5: CD44 isoform 5
CML: Chronic myelogenous leukemia
CSCs: Cancer stem cells
CSF: Cerebrospinal fluid
CTO: Carboxyamidotriazole orotate
DHA: Docosahexaenoic acid
DLL4: Delta-like ligand 4
ECs: Endothelial cells
EMT: Epithelial-mesenchymal transition
ESCRT: Eendosomal sorting complex required for transport
EVs: Extracellular vesicles
GBM: Glioblastoma multiforme
ICAM-1: Intercellular adhesion molecule 1
ILVs: Intraluminal vesicles
ISEV: International society for extracellular vesicles
lncRNA: Long non-coding RNA
MM: Malignant mesothelioma
MSCs: Mesenchymal stem cells
MVBs: Multivesicular bodies
MVs: Microvesicles
PA: Phosphatidic acid
ROS: Reactive oxygen species
SNARE: Soluble *N*-ethylmaleimide-sensitive fusion attachment protein (SNAP) receptors
